# Supplementary material for: Shape-Dependent Toxicity of Silver Nanoparticles on Freshwater Cnidarians
Source: Nanomaterials (Basel). 2022 Sep 7;12(18):3107. doi: 10.3390/nano12183107 (PMC9503847; doi:10.3390/nano12183107)
Supplement: Supplementary file 1 [file nanomaterials-12-03107-s001.zip › nanomaterials-1881825-supplementary.pdf]

Supplementary Materials

Shape-dependent toxicity of silver nanoparticles on freshwater cnidarians.

Auclair, J., Gagné, F.

1. Aquatic Contaminants Research Division, Environment and Climate Change Canada, 105 McGill, Montréal, Québec, Canada H2Y 2E7.

Address all correspondence to: francois.gagne@ec.gc.ca ; joelle.auclair@ec.gc.ca

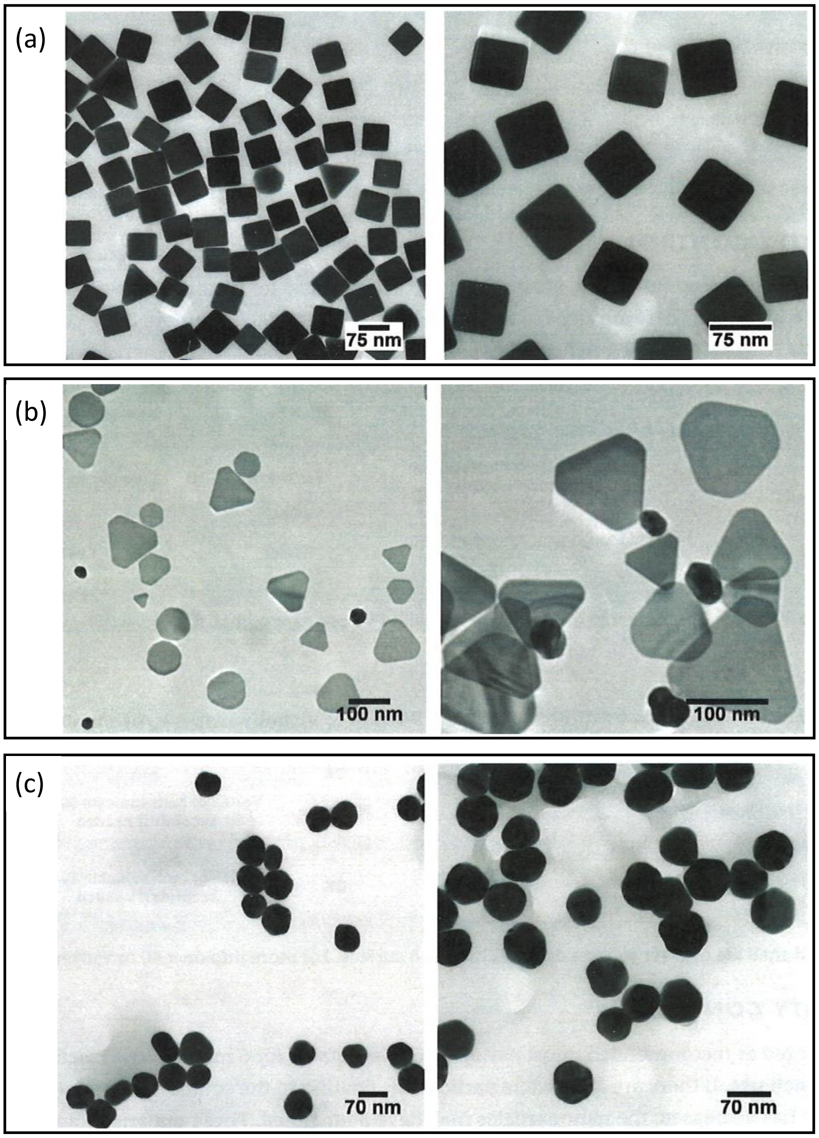

Figure S1. TEM images of (a) cubic, (b) prismatic, and (c) spherical Ag nanoparticles.
